# Treatment preferences among Japanese patients and physicians for epidermal growth factor receptor‐mutant non‐small cell lung cancer

**DOI:** 10.1002/cam4.6777

**Published:** 2024-01-09

**Authors:** Akito Hata, Simon Fifer, Kazuo Hasegawa, Emiko Ando, Mami Kasahara‐Kiritani, Michiko Takahashi, Robyn Ordman, Lili Toh, Akira Inoue

**Affiliations:** ^1^ Division of Thoracic Oncology Kobe Minimally Invasive Cancer Center Kobe Hyogo Japan; ^2^ Community and Patient Preference Research (CaPPRe) Sydney Australia; ^3^ NPO Lung Cancer Patients Association One Step Kanagawa Japan; ^4^ Integrated Market Access Division, Janssen Pharmaceutical K. K. Tokyo Japan; ^5^ Medical Affairs Division, Janssen Pharmaceutical K. K. Tokyo Japan; ^6^ Department of Palliative Medicine Tohoku University School of Medicine Sendai Miyagi Japan

**Keywords:** bioethics, growth factor receptors, informed consent, palliative treatment, social movement against cancer

## Abstract

**Introduction:**

Evidence is limited on preferences of Japanese patients and physicians in treatment for epidermal growth factor receptor (*EGFR*)‐mutant non‐small cell lung cancer (NSCLC). Several oral or intravenous novel agents for *EGFR* exon 20 insertions are under development. The aim of our study was to investigate which attributes of novel treatments influenced selection of oral or intravenous agents among treated patients and treating physicians in Japan.

**Methods:**

The study was designed by board‐certified oncologists, patient representatives, and analytics specialists. Eligible participants completed an online survey with a discrete choice experiment presenting two treatment profiles described by attributes: mode of administration (oral or intravenous); frequency of administration; overall response rate (ORR); average progression‐free survival (PFS); chance of experiencing severe side effects (SEs); mild–moderate gastrointestinal SEs; mild–moderate skin‐related SEs; and patient out‐of‐pocket costs.

**Results:**

Fifty‐four patients (all self‐reported *EGFR*‐mutant) and 74 physicians participated from December 2021 to August 2022. All attributes being equal, there was greater preference for oral administration. However, there was greater preference for intravenous over oral, when ORR and PFS improved by 10% and 1 month, and severe SEs reduced by 10%. Physicians exhibited greater preference for PFS compared to patients (*p* < 0.01). Ranked order of attribute importance was as follows: (1) PFS; (2) ORR; (3) severe SEs, expressed by patients and physicians alike.

**Conclusions:**

Our study revealed Japanese physician and patient preferences in treatment options for *EGFR*‐mutant NSCLC. Compared to the strong preference for a more efficacious drug, the preference of oral versus intravenous revealed a smaller impact.

## Graphical abstract

The purpose of this preference study was to better understand which treatment attributes were most important in selecting novel oral or intravenous treatments among EGFR‐mutant NSCLC patients and treating physicians in Japan using a DCE. The results showed that compared to the strong preference for a more efficacious drug, the preference for oral versus intravenous revealed a smaller impact.

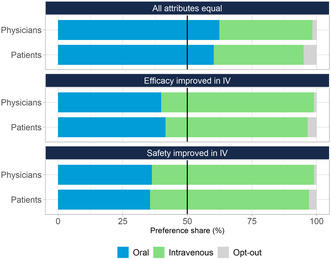

## 1 INTRODUCTION

Lung cancer is a major health problem across the world, with increasing prevalence in Japan (118,971 new cases, 81,820 deaths in 2018^1^). It is the leading cause of cancer‐related death in Japanese males and second among females.^2^ Non‐small cell lung cancer (NSCLC) accounts for 80% of lung cancer diagnoses in Japan.^3^

Approximately one third of NSCLC diagnoses (30%–40%) include epidermal growth factor receptor (*EGFR*) gene mutations, considered to be the “driver” gene, higher among Asian patients.^3^, ^4^ Common *EGFR* mutations such as Exon 19 deletion (Del19) and Exon 21 Leu858Arg substitution (L858R) account for more than 80% of all *EGFR* mutations^5^ and are effectively treated by tyrosine kinase Inhibitors (TKIs)^6^, ^7^ which have been shown to improve patient tolerability and overall survival compared to standard chemotherapy.^3^, ^4^

However, commercially available first to third generation EGFR‐TKIs have demonstrated limited efficacy against more uncommon *EGFR* mutation subtypes such as *EGFR* Exon 20 insertion (*EGFR* ex20in) mutation,^8^, ^9^ which represents 3%–9% of all *EGFR* mutations.^10^, ^11^ Consequently, there is a lack of efficient target therapy options against *EGFR* ex20in NSCLC as EGFR‐TKIs are not a recommended treatment option according to current treatment guidelines in Japan.^12^ Instead, the current standard of care for patients with *EGFR* ex20in NSCLC is conventional cytotoxic chemotherapy, which involves a higher level of toxicity compared to other treatments such as EGFR‐TKIs.^12^

Novel *EGFR* inhibitors are under development to provide more treatment options for patients with *EGFR*‐mutated NSCLC—including those with *EGFR* ex20in mutation. Inhibitors include amivantamab (JNJ372)^13^ and mobocertinib (TAK788)^14^ that vary across several attributes including mode of administration (oral or intravenous), frequency of administration, efficacy, risk of side effects, and out‐of‐pocket cost.

Although there is existing preference research for NSCLC treatments,^15^, ^16^, ^17^, ^18^ evidence of Japanese patient and physician preferences that consider attribute profiles of novel *EGFR* ex20in mutation treatments have yet to be explored. Since patients living with *EGFR*‐mutated NSCLC and treating physicians represent primary decision‐makers in treatment choice, it is critical to understand what treatment attributes are important to them. In addition, describing similarities/differences of patients and physicians preferences in their decision‐making would be clinically important to foster shared decision‐making through deeper discussions.^19^, ^20^

The current study aimed to investigate preferences for novel NSCLC treatments among patients with *EGFR*‐mutated NSCLC and treating physicians. Specifically: (i) treatment attributes that patients and physicians value and (ii) the similarities and/or differences between patient and physician treatment attribute preferences.

## 2 MATERIALS AND METHODS

### 2.1 Participants

Participants were invited to take part in a 20‐min online survey presented in Japanese by global specialist healthcare online panel companies with local teams in Japan.

### 2.2 Inclusion criteria

Participants 18 years or older, fluent in Japanese were eligible if they were diagnosed with NSCLC, were in stage 3 or 4 of the disease, and were considered to have NSCLC *EGFR*‐mutated status through either (a) self‐reporting a positive test result for *EGFR* or (b) indicated they have received EGFR‐tyrosine kinase inhibitor (EGFR‐TKI). Patients with *EGFR* ex20in NSCLC as well as patients with *EGFR*‐mutated NSCLC were included in our study because an exclusive sample of *EGFR* ex20in NSCLC patients would make it difficult to achieve a statistically powerful sample size, given the rarity of this mutation. While recruitment efforts prioritized *EGFR* ex20in NSCLC patients, the sample included broader *EGFR*‐mutated NSCLC patients. Physicians must be in a related specialty (oncology, cancer drug specialist, cancer treatment certified physician, respiratory, and internal treatment), have experience treating NSCLC *EGFR*‐mutated patients, and have prescribed chemotherapy, EGFR‐TKI, or other targeted therapies to be included in the analysis.

### 2.3 Exclusion criteria

Participants were excluded if they were employees of a pharmaceutical company. They were also excluded if their responses were not considered to be genuine through criteria such as selecting clinically invalid treatments (for patients), giving poor open‐text responses, failing attention questions with an obvious correct response (e.g., “If you are paying attention, please select ‘Moderately disagree’ below”), completing the survey too quickly (less than 7 min), or were duplicates. Additionally, participants were excluded if they had a poor understanding of the Discrete Choice Experiment (DCE) (a rating of less than six on a scale from 1 [“Did not understand the scenarios at all”] to 10 [“Completely understood the scenarios”]).

### 2.4 Assessing treatment preferences

In order to understand patient and physician preferences, the current study conducted a DCE to determine the relative importance individuals place on each attribute and how that impacts decision‐making via experimental design and modeling. DCEs are a prominent and reliable method used in fields such as health technology assessments and economics,^21^, ^22^, ^23^ and treatment preferences of NSCLC^15^ to understand and model trade‐offs and preferences revealed by choices people make.

In a DCE, participants respond to a series of hypothetical choice scenarios, each presenting competing alternatives made up of several attributes. Levels of attributes vary across the series of choice scenarios based on experimental design. In each scenario, participants are asked to choose their preferred alternative. Through this, participants trade‐off features when selecting an alternative that maximizes their “utility”. How participants change responses across various choice scenarios are observed, and the importance placed on these attributes can then be inferred via statistical modeling. The current study improves on previous DCE studies (e.g.,^17^, ^24^) by including an “opt out” choice in each scenario to capture real choices of participants. This way, participants are not forced to choose either alternative, thereby reducing bias in the results.

### 2.5 Study design

The current study utilized a cross‐sectional, noninterventional design with a DCE to understand treatment preferences of patients with *EGFR*‐mutated NSCLC and treating physicians. A four‐stage approach was used to design and conduct the DCE in a quantitative online survey. The study was designed by board‐certified oncologists, patient representatives, and analytics specialists.

### 2.6 Development of DCE attributes and levels

The initial three stages were used to inform attributes and levels of the DCE that was conducted in the fourth stage. First, a rapid literature review on existing preference and market research studies for NSCLC was conducted to identify relevant treatment preference attributes. Second, in‐depth qualitative interviews were conducted with two patients with *EGFR*‐mutated NSCLC and three physicians treating a mix of common NSCLC and *EGFR* ex20 mutated NSCLC to explore and understand key attributes and levels. Third, results of the qualitative interviews were discussed with a steering committee which included patient representatives and NSCLC‐treating physicians to agree on DCE attributes and levels that were clinically meaningful to them and expressed in plain language to improve readability for patient participants. For example, “average progression‐free survival” was used to describe PFS instead of “median progression‐free survival”.

The DCE presented two treatment alternatives; an oral treatment and an intravenous (IV) treatment. The final attributes to describe the two treatment alternatives were as follows: frequency of administration, overall response rate (ORR), average progression‐free survival (PFS), chance of experiencing mild–moderate gastrointestinal side effects, chance of experiencing mild–moderate skin‐related side effects, chance of experiencing any severe side effects, and yearly patient out‐of‐pocket costs. A full list of attributes, their descriptions, and range of levels is presented in Table 1.

**TABLE 1:** DCE attributes, levels, and their descriptions.

Attributes	Attribute description	Levels	Level description
Frequency of administration	How frequently the treatment is taken by you or given to you.	Once every week (IV)	Treatment is given intravenously (through a thin needle or tube into a vein) once a week.
		Once every 2 weeks (IV)	Treatment is given intravenously (through a thin needle or tube into a vein) once every 2 weeks.
		Once every 3 weeks (IV)	Treatment is given intravenously (through a thin needle or tube into a vein) once every 3 weeks.
		Once a month (IV)	Treatment is given intravenously (through a thin needle or tube into a vein) once a month.
		Two times a day (Oral)	Treatment is taken orally (as a pill or tablet to be swallowed by mouth) twice a day.
		One time a day (Oral)	Treatment is taken orally (as a pill or tablet to be swallowed by mouth) once a day.
Overall response rate (ORR)	Chance of tumor size decrease of at least 30% in volume on a CT scan.	10% (10 in 100)	10% of patients (10 in 100) see a response on this treatment.
		20% (20 in 100)	20% of patients (20 in 100) see a response on this treatment.
		30% (30 in 100)	30% of patients (30 in 100) see a response on this treatment.
		40% (40 in 100)	40% of patients (40 in 100) see a response on this treatment.
		50% (50 in 100)	50% of patients (50 in 100) see a response on this treatment.
		60% (60 in 100)	60% of patients (60 in 100) see a response on this treatment.
Average progression‐free survival (PFS)	The average time a person on this treatment is expected to live without progression or worsening of disease, based on clinical trial information.	4 months	Treatment prevents disease from progressing or getting worse for 4 months, on average.
		8 months	Treatment prevents disease from progressing or getting worse for 8 months, on average.
		12 months	Treatment prevents disease from progressing or getting worse for 12 months, on average.
		16 months	Treatment prevents disease from progressing or getting worse for 16 months, on average.
		20 months	Treatment prevents disease from progressing or getting worse for 20 months, on average.
		24 months	Treatment prevents disease from progressing or getting worse for 24 months, on average.
Chance of experiencing mild–moderate gastrointestinal side effects	Chance of treatment causing mild–moderate gastrointestinal side effects, for example, mild–moderate diarrhea, nausea, vomiting, and bloating. These are manageable enough that you do not want to stop the treatment.	5% (5 in 100)	5% of patients (5 in 100) on this treatment experience mild–moderate skin‐related side effects.
		35% (35 in 100)	35% of patients (35 in 100) on this treatment experience mild–moderate skin‐related side effects.
		65% (65 in 100)	65% of patients (65 in 100) on this treatment experience mild–moderate skin‐related side effects.
		95% (95 in 100)	95% of patients (95 in 100) on this treatment experience mild–moderate skin‐related side effects.
Chance of experiencing mild–moderate skin‐related side effects	Chance of treatment causing mild–moderate skin‐related side effects, for example, mild–moderate rash, maculopapular rash, acne, and dry skin. These are manageable enough that you do not want to stop the treatment.	5% (5 in 100)	5% of patients (5 in 100) on this treatment experience mild–moderate skin‐related side effects.
		35% (35 in 100)	35% of patients (35 in 100) on this treatment experience mild–moderate skin‐related side effects.
		65% (65 in 100)	65% of patients (65 in 100) on this treatment experience mild–moderate skin‐related side effects.
		95% (95 in 100)	95% of patients (95 in 100) on this treatment experience mild–moderate skin‐related side effects.
Chance of experiencing any severe side effects	Chance of treatment causing severe side effects. Can include any side effects. You need to be hospitalized or stop the treatment.	1% (1 in 100)	1% of patients (1 in 100) on this treatment experience ANY severe side effects.
		5% (5 in 100)	5% of patients (5 in 100) on this treatment experience ANY severe side effects.
		10% (10 in 100)	10% of patients (10 in 100) on this treatment experience ANY severe side effects.
		20% (20 in 100)	20% of patients (20 in 100) on this treatment experience ANY severe side effects.
Yearly cost	Yearly treatment cost that you need to pay.	¥100,000	Cost to be paid out of pocket is ¥100,000 annually.
		¥500,000	Cost to be paid out of pocket is ¥500,000 annually.
		¥1,000,000	Cost to be paid out of pocket is ¥1,000,000 annually.
		¥1,500,000	Cost to be paid out of pocket is ¥1,500,000 annually.
		¥2,000,000	Cost to be paid out of pocket is ¥2,000,000 annually.

*Note*: The United States dollar (USD) to Japanese yen (JPY) conversion rate was approximately 1 USD to 134 JPY at the end of data collection.

### 2.7 DCE experimental design

The DCE experimental design is a matrix of values used to determine which levels are shown in which choice scenarios. A Bayesian efficient design requires the provision of prior information about the parameters (commonly referred to as priors). Our study used naïve priors. This was done using Ngene version 1.3 software.^25^ The experimental design consisted of six blocks with 10 choice scenarios each (60 scenarios in total). That is, each participant was randomly assigned to one block and responded to 10 choice scenarios (see Figure, Data S1, which presents an example patient DCE scenario in English).The online quantitative survey including the DCE was hosted and programmed using the Forsta survey platform.^26^

### 2.8 Statistical analysis

To allow for preference heterogeneity between participants, a mixed multinomial logit (MMNL) model was used to estimate DCE data. Patient and physician DCE data were pooled to allow for a larger overall sample size and to check for differences between patients and physicians for each parameter. Attributes such as ORR, average PFS, and yearly cost were coded as continuous (linear) while the remaining attributes such as frequency of administration, chance of any severe side effects, chance of mild–moderate gastrointestinal side effects, and chance of mild–moderate skin‐related side effects were categorically coded. The MMNL estimated parameters for each level to determine whether and how much each value predicted treatment choice (see Formulae, Data S2, which specifies utility equations used). DCE data were modeled using the econometric software, Nlogit version 6.^27^ The relative importance of each attribute was calculated by finding the maximum difference in utility between the attribute's levels and expressing it as a percentage of the sum of all maximum differences (such that importance of all attributes sum up to 100%).

### 2.9 Measures of background variables

Age, gender, and geographical settings were collected as demographic variables from all participants. Additionally, patients annual household income was collected, while physicians were asked about their specialty and the region they currently practice in. Lung cancer stage at time of data collection and mutation status were reported as patient disease background variables. Additionally, experience of receiving intravenous treatment and prior targeted treatments were collected from patients as treatment background variables.

## 3 RESULTS

**FIGURE 1:**
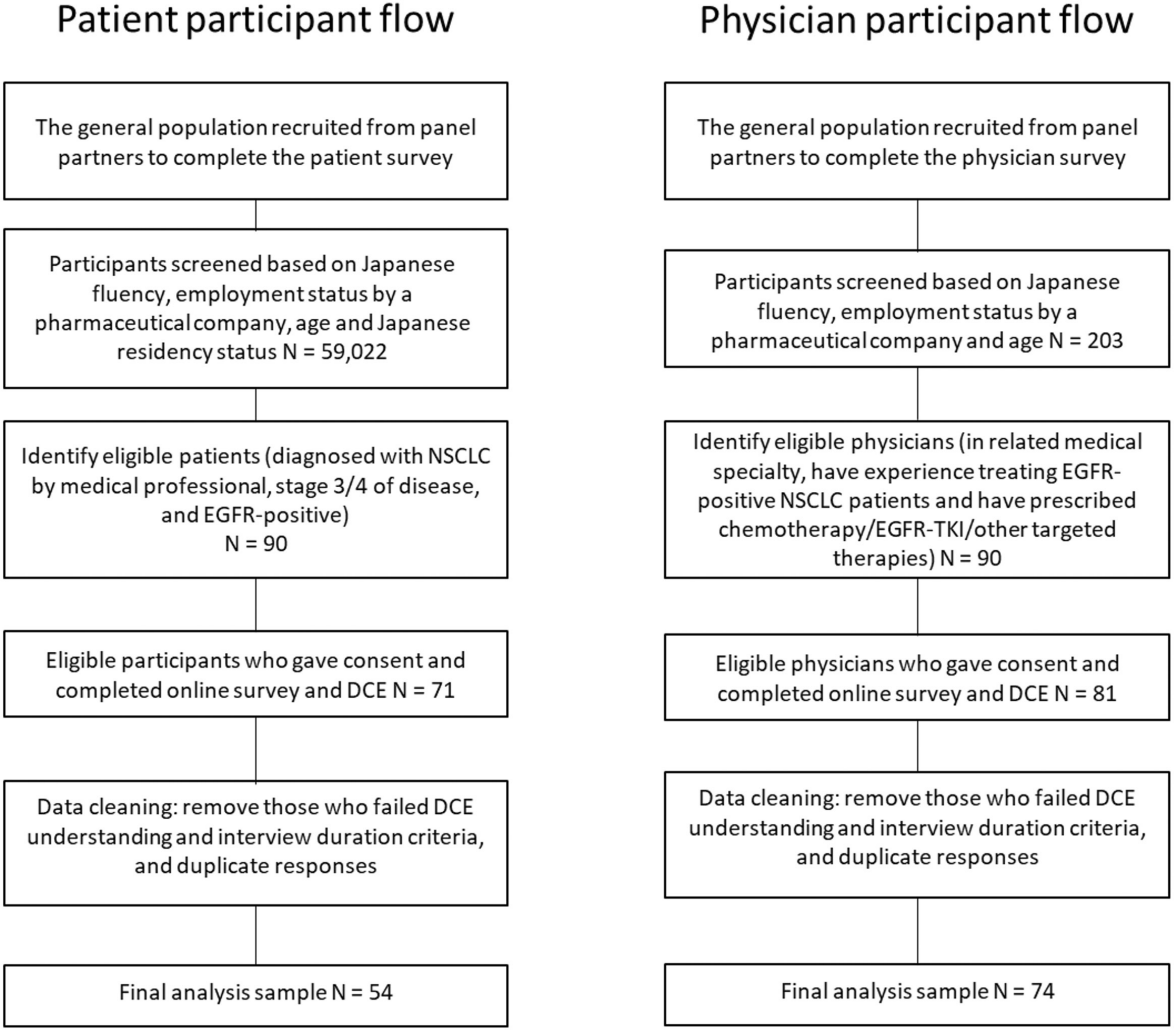
Patient and physician participant flowchart.

A total of 128 eligible participants completed the survey (*n* = 54 patients, *n* = 74 physicians). The participant flowchart for both patients and physicians is presented in Figure 1. All patients reported having stage 3b or 4 NSCLC at the time of the survey and were considered to be *EGFR*‐positive based on a self‐reported positive test result (*n* = 49) or indicated they had received targeted therapy against *EGFR* mutation (*n* = 5). A majority of the patient sample had common *EGFR*‐mutations (65%), approximately one third had other mutations or an unknown status, and a small number had Exon20 in status (6%). In addition, approximately half of the patient sample had received IV treatment for NSCLC. There were slightly more female patients. The age of patients ranged from 20 to 80 years old (median age group is 51–60 years old). Patients' and physicians' background data are summarized in Table 2.

**TABLE 2:** Summary of patients' and physicians' background data.

Demographic characteristic	Total	% Sample
**Patients** (***N*** = **54**)		
Gender		
Female	30	56%
Male	24	44%
NSCLC stage at time of survey		
Stage 3b	21	39%
Stage 4	33	61%
Annual household income (JPY)		
Less than 5,000,000	18	33%
5,000,000‐10,000,000	24	44%
More than 10,000,000	7	13%
Prefer not to answer	5	9%
Mutation status		
*EGFR* mutation	54	100%
Common (Ex19 del, L858R)	35	65%
Exon 20 ins	3	6%
Other and unknown	16	30%
History of IV treatment		
Received IV treatment for NSCLC	31	57%
None	23	43%
Prior treatment (multichoice)		
3rd generation EGFR‐TKI	42	78%
1st and 2nd generation EGFR‐TKIs	29	54%
Immune‐checkpoint inhibitors	13	24%
Other	6	11%
**Physicians** (***N*** = **74**)		
Gender		
Female	8	11%
Male	65	88%
Unknown	1	1%
Specialty		
Medical oncologist	10	14%
Respiratory physician	64	86%
Geography		
Urban	55	74%
Rural	19	26%

*Note*: The United States Dollar (USD) to Japanese yen (JPY) conversion rate was approximately 1 USD to 134 JPY at the end of data collection.

### 3.1 Patients' and physicians' treatment attributes as predictors of choice

Attributes that significantly predicted choice of treatments were frequency of administration, ORR, average PFS, chance of experiencing mild–moderate gastrointestinal side effects, chance of experiencing mild–moderate skin‐related side effects, chance of experiencing any severe side effects, and yearly patient out‐of‐pocket costs. Only frequency of administration for the oral treatment did not emerge as a significant predictor of treatment choice relative to other attributes. Table 3 presents the model parameters and coefficients. Comparing patients' and physicians' treatment preferences, they were not statistically different except for average PFS, which was valued more strongly by physicians for both oral and IV treatments.

**TABLE 3:** DCE model output.

Code	Parameter (attribute and levels)	Coefficient	Sig.	SE	T‐Ratio	Interpretation
Random parameters						
RESPC	Overall response rate (ORR)—decrease in tumor size (continuous)	0.06129	***	0.00723	8.47	Preference is higher with higher response rate/efficacy
PFSC	Average progression‐free survival (continuous)	0.33117	***	0.03512	9.43	
GI	95% chance of mild–moderate gastrointestinal side effects (reference category)	−0.63101	ref			Preference is higher with lower chance of gastrointestinal and severe side effects
GI_12	35% or 65% chance of mild–moderate gastrointestinal side effects	0.11771	ns	0.09362	1.26	
GI_3	5% chance of mild–moderate gastrointestinal side effects	0.5133	***	0.12423	4.13	
SEV	20% chance of severe side effects (reference category)	−1.48835	ref			
SEV_1	10% chance of severe side effects	−0.13927	ns	0.1291	−1.08	
SEV_2	5% chance of severe side effects	0.66959	***	0.12576	5.32	
SEV_3	1% chance of severe side effects	0.95803	***	0.16613	5.77	
COSTC	Yearly cost (continuous)	−0.00026	*	0.00014	−1.88	Preference is lower when cost is higher
FREQ	Frequency of IV administration (once every week) (reference category)	−0.3439	ref			Preference is higher when IV administration is less frequent
FREQ_12	Frequency of IV administration (once every 2 or 3 weeks)	0.01081	ns	0.12814	0.08	
FREQ_3	Frequency of IV administration (once a month)	0.33309	**	0.15563	2.14	
Nonrandom parameters						
ORAL	Oral treatment	−1.04158	***	0.33237	−3.13	Greater preference for oral over IV (relative to neither)
IV	Intravenous (IV) treatment	−1.24884	***	0.35244	−3.54	
SK	95% chance of mild–moderate skin‐related side effects (reference)	−0.19127	ref			Preference is higher with lower chance of skin side effects
SK_1	65% chance of mild–moderate skin‐related side effects	−0.19091	*	0.10685	−1.79	
K_23	35% or 5% chance of mild–moderate skin‐related side effects	0.38218	***	0.09954	3.84	
Heterogeneity in the means between patients and physicians						
PFSC:PTS	Average progression‐free survival (interaction)	−0.11417	***	0.03677	−3.11	PFS is more important for physicians than patients (lower)

*Note*: The magnitude of the coefficient indicates the strength of the predictive value of each attribute. Negative and positive values of the coefficient indicate the direction of the relationship (i.e., a more positive value indicates greater preference). *** Significant at 1% level; ** Significant at 5% level; *Significant at 10% level; ns = not significant; ref = reference category.

### 3.2 Preference shares

**FIGURE 2:**
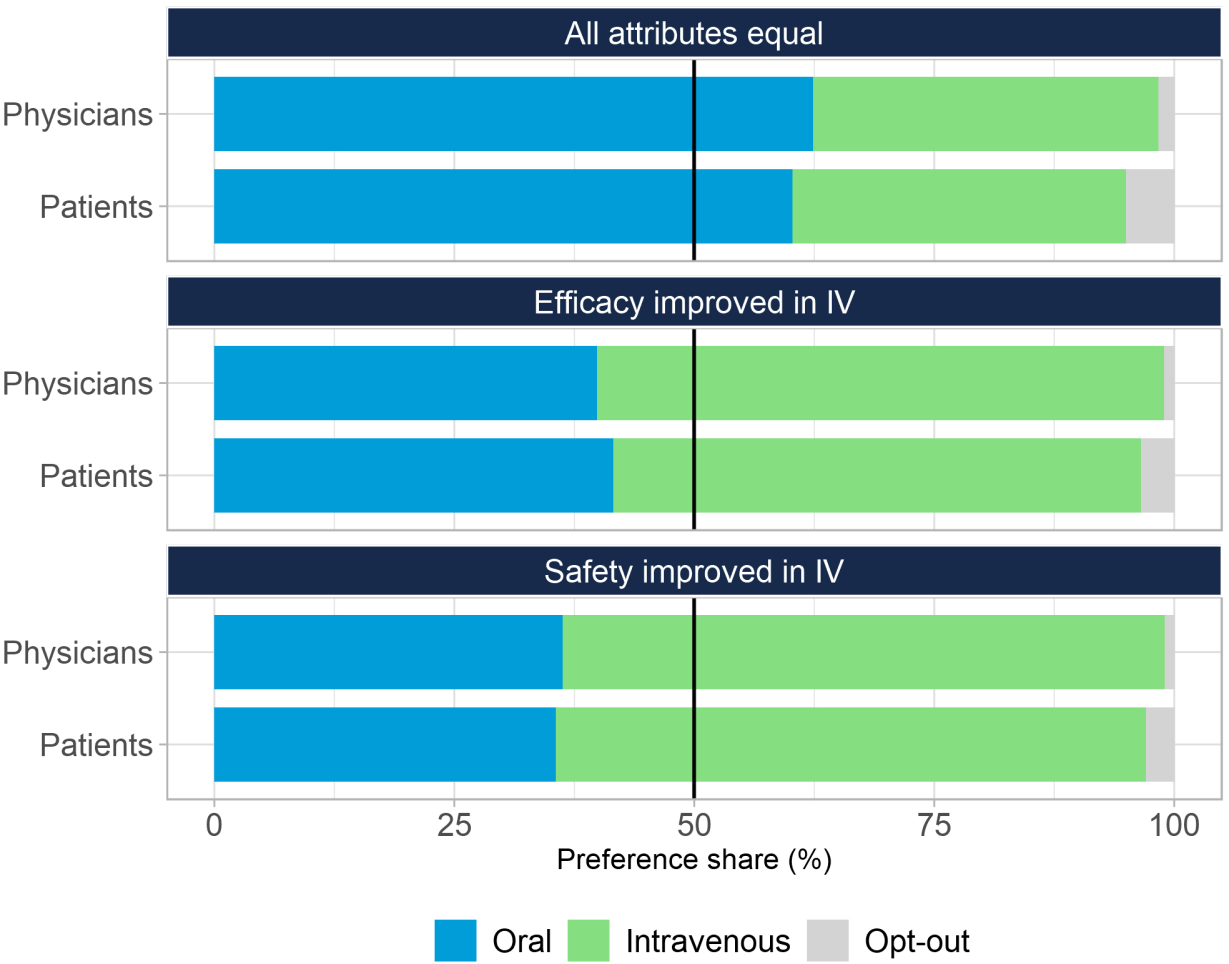
Patient and physician preference shares. Preference shares should be viewed in the context of the levels specified above; when all attributes for oral and IV alternatives were held equal, when efficacy is improved for the IV alternative (ORR is improved by 10% and average PFS extended by 1 month), when safety is improved for the IV alternative (chance of any severe side effects reduced to 1%) for the IV alternative.

Model parameters were used to simulate preference shares. When all attributes for the oral and IV alternatives were held equal, there is a slight preference for an oral alternative compared to IV or the opt out (oral = 60.28%; IV = 34.74% for patients; oral = 62.40%; IV = 35.96% for physicians). When efficacy is improved (ORR improved by 10% and average PFS extended by 1 month) for the IV alternative, there is a greater preference for the IV alternative than the oral or opt out (oral = 41.60%; IV = 54.97% for patients; oral = 39.88%; IV = 59.07% for physicians). When safety is improved (chance of any severe side effects reduced to 1%) for the IV alternative, there is a greater preference for the IV alternative than the oral or opt out (oral = 35.60%; IV = 61.46% for patients; oral = 36.33%; IV = 62.72% for physicians). These are illustrated in Figure 2. Other example simulations of preference shares are displayed below in Table 4 to demonstrate the predictive relationship of ORR, and PFS on treatment choice based on the model.

**TABLE 4:** Example simulations of preference shares based on model coefficients.

Change from base levels	Oral preference share	IV preference share
No change, all equal		
Patient	60.28%	34.74%
Physician	62.4%	35.96%
Increase ORR by 8% for IV		
Patient	49.42%	46.5%
Physician	50.83%	47.83%
Increase ORR by 9% for IV		
Patient	48.01%	48.03%
Physician	49.34%	49.36%
Increase ORR by 10% for IV		
Patient	46.59%	49.56%
Physician	47.85%	50.89%
Increase ORR by 11% for IV		
Patient	45.18%	51.09%
Physician	46.35%	52.42%
Increase PFS by 1 month for IV		
Patient	55.6%	39.81%
Physician	54.68%	43.88%
Increase PFS by 2 months for IV		
Patient	50.71%	45.1%
Physician	46.64%	52.13%
Increase PFS by 3 months for IV		
Patient	45.71%	50.51%
Physician	38.72%	60.26%

### 3.3 Patients' and physicians' relative attribute importance

**FIGURE 3:**
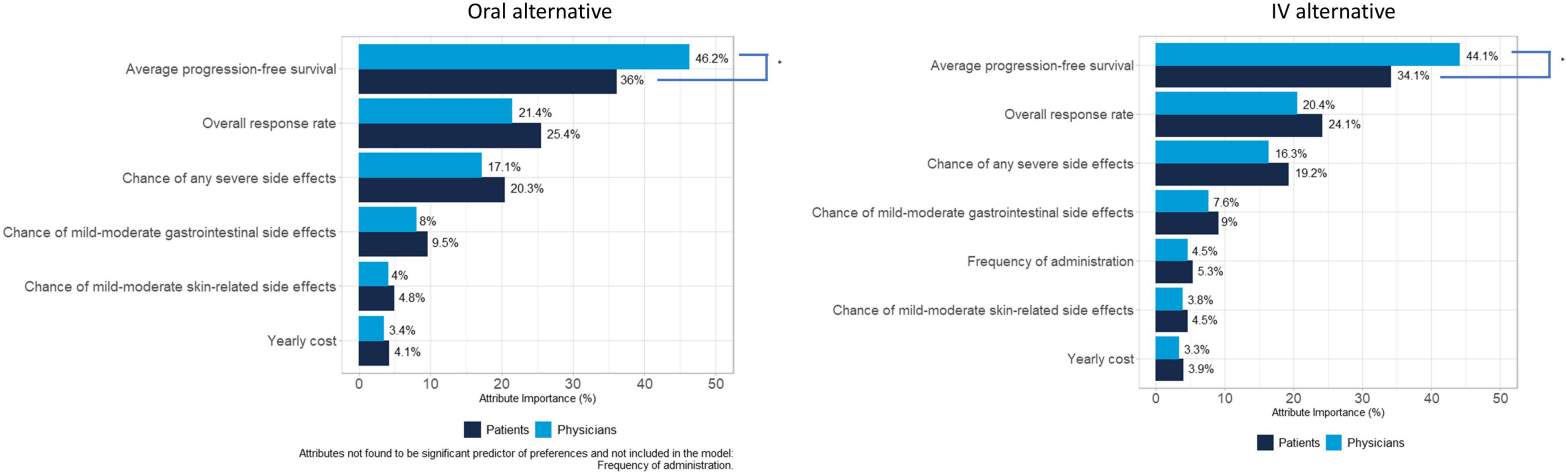
Patient and physician attribute importance of the oral and IV alternative. Percentages within each of the oral and IV alternative graphs sum up to 100. Asterisks represent significant differences between patients and physicians at *p* < 0.05. Attribute importance are represented by dark blue bars for patients and light blue bars for physicians.

Figure 3 illustrates the relative importance of each attribute of the oral and IV alternative for both patients and physicians. Patients and physicians were aligned on order of attribute importance across alternatives (i.e., oral/IV). Average PFS, ORR, and chance of any severe side effects were the most important attributes. Among other side effects, chance of mild–moderate GI side effects were perceived to be relatively more important than mild–moderate skin‐related side effects for both patients and physicians across both alternatives. Although physicians place significantly more importance on average PFS, patients place more importance on all remaining attributes compared to physicians.

## 4 DISCUSSION

To the best of our knowledge, this is the first study to examine patient and physician preferences of *EGFR*‐mutated NSCLC treatments in a Japanese population. Our study was a collaboration with two patient representative authors who were involved from study conceptualization to interpretation of results and manuscript development in an effort to foster better patient‐centric research in Japan. Their contributions enhanced the study by improving readability of the survey and manuscript from a patient's perspective. While previous preference research^28^ focused solely on patients, we extend existing findings by focusing on both patients and treating physicians, who both play vital roles in treatment decision‐making. Results demonstrate what patients and physicians in Japan are willing to trade‐off for higher efficacy and safety, and the level of importance patients placed on attributes compared to physicians. These insights help improve physicians' and patients' mutual understanding of treatment choice to facilitate shared decision‐making.

All attributes were significant predictors of treatment choice except for frequency of administration for the oral treatment. This does not indicate that frequency of administration does not influence treatment choice, but that the effect may not strongly predict treatment choice relative to the other attributes. When comparing patients' and physicians' treatment preferences, they were similar and differed only on PFS which physicians valued significantly more than patients for both treatment alternatives. Perhaps patients prioritized this attribute to a lesser extent because of their lived experience with other treatment attributes that were considered alongside PFS, while physicians prioritized the extension of PFS as a treatment outcome. This pattern of results is comparable to previous findings among NSCLC patients and treating physicians in China, that physicians were willing to trade‐off on more than double the cost of treatment to extend PFS compared to patients^17^, ^24^—showing that physicians tend to focus on treating the disease in isolation which may not completely align with patient preferences. Differences in patient and physician perspectives highlight the importance of shared decision‐making for patient‐centric treatment.

When preference shares were simulated from the model estimates, there was a slight preference for the oral alternative compared to the IV or opt out when all attributes were held equal. This could be due to the convenience of having an oral treatment compared to the IV which is more invasive and time‐consuming. Findings suggest that patients and physicians are willing to trade the convenience of an oral alternative for an improvement in efficacy and/or safety. This pattern reflects previous findings among patients that PFS was regarded as the most important attribute in *EGFR*‐mutated NSCLC treatments, and mode of administration (oral or IV) was the least important among other attributes depicting side effects (e.g., rash, diarrhea, nausea, and vomiting).^15^ Differences in the magnitude of attribute importance, particularly the most important attributes (PFS, ORR, and severe side effects) relative to other attributes drove patient and physician preference choice.

Findings on attribute importance reflect previous NSCLC treatment preference studies where efficacy (PFS and ORR) was the most influential of the attributes presented.^15^, ^17^, ^29^ In addition, our study found that physicians placed more importance on PFS while patients tended to hold more importance on other remaining attributes compared to physicians. We attribute this to the lived experience patients have to consider when making treatment choices compared to physicians.^17^, ^24^

Among side effects, previous studies found that severe side effects such as severe fatigue were considered the most influential side effects risk,^18^, ^30^ which is consistent with our findings. Our finding that chance of gastrointestinal side effects were considered to be relatively more important than chance of skin‐related side effects were also consistent with previous findings where chance of gastrointestinal side effects such as nausea/vomiting and diarrhea were considered more influential treatment attributes^16^, ^24^, ^31^ compared to chance of skin‐related side effects such as rash.^18^, ^24^, ^30^

Findings should be interpreted in consideration of the study's limitations. First, is the selection of an eligible population. Although the study examined preferences on novel EGFR‐mutated NSCLC treatments including those effective to *EGFR* ex20in, the sample included patients with broader *EGFR*‐mutated NSCLC as it was too difficult to attain an exclusive sample of patients with NSCLC *EGFR* ex20in. Management of patients with Exon 20 insertion is quite different from that in patients with Exon 19 deletion and Exon 21 mutation. Therefore, it may not be reasonable to analyze those patients at the same time in our study if statistical power is large enough. However, the inclusion of broader *EGFR*‐mutated NSCLC patients allowed us to capture preferences from those who had experience receiving EGFR‐TKIs, thus yielding more accurate preferences of decision‐makers considering novel EGFR‐TKIs. Second, the patient sample size for the current study was relatively small to draw a clinically solid conclusion. However, demographic and background characteristics were similar to previous studies that recruited a larger sample.^32^ Similarly, it would be interesting to examine preference shares and attribute importance of patient subgroups (e.g., prior IV vs. non‐prior IV patients). However, the small sample size of these subgroups would not yield meaningful results due to the lack of statistical power. Future studies with access to greater resources, particularly with recruitment, are encouraged to explore differences between these subgroups. Third, patients were included based on self‐reported diagnosis and treatment rather than objective measures such as official health records. In spite of these limitations, findings share valuable insight on novel *EGFR* ex20in mutation treatment profiles from patients with *EGFR*‐mutated NSCLC and treating physicians.

In conclusion, the current study revealed that patients and physicians were aligned on order of attribute importance of *EGFR*‐mutated NSCLC treatments. Physicians placed more importance on PFS than patients, while patients placed more importance on all remaining attributes (relative to PFS) than physicians. Findings shed light on what attributes patients and physicians value in treatment profiles of novel NSCLC *EGFR* treatments including *EGFR* ex20in treatments currently under development, and the importance of shared decision‐making.

## AUTHOR CONTRIBUTIONS

**Akito Hata:** Conceptualization (equal); methodology (equal); writing – review and editing (equal). **Simon Fifer:** Conceptualization (equal); formal analysis (equal); investigation (equal); methodology (equal); supervision (equal); writing – review and editing (equal). **Kazuo Hasegawa:** Conceptualization (equal); methodology (equal); writing – review and editing (equal). **Emiko Ando:** Conceptualization (equal); methodology (equal); writing – review and editing (equal). **Mami Kasahara‐Kiritani:** Conceptualization (equal); funding acquisition (equal); methodology (equal); project administration (equal); supervision (equal); writing – review and editing (equal). **Michiko Takahashi:** Conceptualization (equal); funding acquisition (equal); methodology (equal); writing – review and editing (equal). **Robyn Ordman:** Conceptualization (equal); investigation (equal); methodology (equal); project administration (equal); writing – review and editing (equal). **Lili Toh:** Data curation (equal); investigation (equal); software (equal); visualization (equal); writing – original draft (equal). **Akira Inoue:** Conceptualization (equal); methodology (equal); supervision (equal); writing – review and editing (equal).

## FUNDING INFORMATION

This work was funded by Janssen Pharmaceutical K.K.

## CONFLICT OF INTEREST STATEMENT

AH reports receiving honoraria for lectures and presentations for Eli Lilly, Chugai, Pfizer, AstraZeneca, Boehringer‐Ingelheim, Taiho, and MSD, and funding MSD, Eli Lilly, Boehringer‐Ingelheim, AstraZeneca, Taiho, and Chugai. KH and EA are both patient partners from NPO Lung Cancer Patients Association One Step, MK and MT are both employees from Janssen Pharmaceutical K.K. AI reports receiving honoraria for lectures and presentations for AstraZeneca and Eli Lilly. SF, RO, and LT are from CaPPRe who were contracted by Janssen Pharmaceutical to design the experiment, provide data management, conduct the analysis, and develop the manuscript.

## ETHICS STATEMENT

Approval of the research protocol by an Institutional Reviewer Board: The current study was approved by the MINS Institutional Review Board^33^ based in Japan (JS2021‐29266) and was carried out in accordance with the Declaration of Helsinki.

## INFORMED CONSENT

Informed consent was obtained online by participants checking a box that they have read the Participant Information Sheet and Consent Form and consent to participate.

## Supporting information


Appendix S1

## Data Availability

Research data are not shared.
