# Supplementary material for: Treatment preferences among Japanese patients and physicians for epidermal growth factor receptor‐mutant non‐small cell lung cancer
Source: Cancer Med. 2024 Jan 9;13(1):e6777. doi: 10.1002/cam4.6777 (PMC10807555; doi:10.1002/cam4.6777)
Supplement: Supplementary file 1 — Appendix S1 [file CAM4-13-e6777-s001.docx]

## Supplemental Data 1:

## Example patient DCE scenario in English.

##
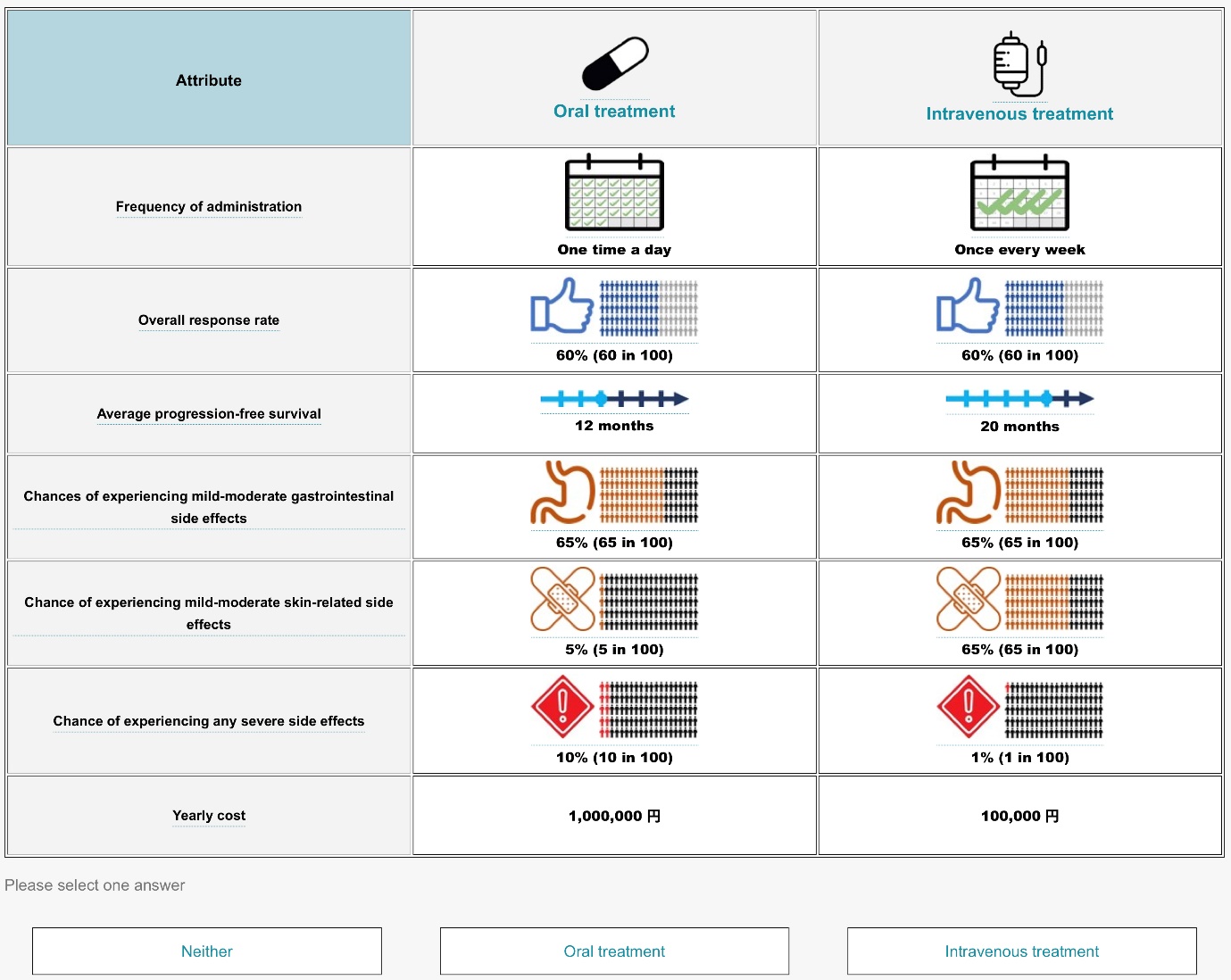


## Supplemental Data 2:

## Utility equations specified in the mixed multinomial logit model.

### Patients

$$U_{ORAL}=\beta_{ORAL}+\beta_{RESP}x_{RESP}+\beta_{PFS}x_{PFS}+\beta_{GI12}x_{GI12}+\beta_{GI3}x_{GI3}+\beta_{SK1}x_{SK1}+\beta_{SK23}x_{SK23}+\beta_{SEV1}x_{SEV1}+\beta_{SEV2}x_{SEV2}+\beta_{SEV3}x_{SEV3}+\beta_{COST}x_{COST}+\epsilon_{ORAL}$$

$$U_{IV}=\beta_{IV}+\beta_{FREQ12}x_{FREQ12}+\beta_{FREQ3}x_{FREQ3}+\beta_{RESP}x_{RESP}+\beta_{PFS}x_{PFS}+\beta_{GI12}x_{GI12}+\beta_{GI3}x_{GI3}+\beta_{SK1}x_{SK1}+\beta_{SK23}x_{SK23}+\beta_{SEV1}x_{SEV1}+\beta_{SEV2}x_{SEV2}+\beta_{SEV3}x_{SEV3}+\beta_{COST}x_{COST}+\epsilon_{IV}$$

### Physicians

$$U_{ORAL}=\beta_{ORAL}+\beta_{RESP}x_{RESP}+\beta_{PFS}x_{PFS}+\beta_{GI12}x_{GI12}+\beta_{GI3}x_{GI3}+\beta_{SK1}x_{SK1}+\beta_{SK23}x_{SK23}+\beta_{SEV1}x_{SEV1}+\beta_{SEV2}x_{SEV2}+\beta_{SEV3}x_{SEV3}+\beta_{COST}x_{COST}+\epsilon_{ORAL}$$

$$U_{IV}=\beta_{IV}+\beta_{FREQ12}x_{FREQ12}+\beta_{FREQ3}x_{FREQ3}+\beta_{RESP}x_{RESP}+\beta_{PFS}x_{PFS}+\beta_{GI12}x_{GI12}+\beta_{GI3}x_{GI3}+\beta_{SK1}x_{SK1}+\beta_{SK23}x_{SK23}+\beta_{SEV1}x_{SEV1}+\beta_{SEV2}x_{SEV2}+\beta_{SEV3}x_{SEV3}+\beta_{COST}x_{COST}+\epsilon_{IV}$$
